# 4043 Saliva microRNA for pediatric concussion assessment

**DOI:** 10.1017/cts.2020.342

**Published:** 2020-07-29

**Authors:** Steven Hicks, Jayson Loeffert, Andrea Loeffert, Cayce Onks, Kevin Zhen, Raymond Kim, Robert Olympia

**Affiliations:** 1 Penn State Clinical and Translational Science Institute; 2 Penn State College of Medicine

## Abstract

OBJECTIVES/GOALS: There is no objective, biologic tool to detect concussion or guide clinical management. We previously showed that saliva microRNA (miRNA) levels differ in children with concussion and may predict symptom duration. The purpose of this study was to validate the utility of saliva miRNA and define longitudinal trends during the recovery period. METHODS/STUDY POPULATION: We collected concussion symptom burden (SCAT-5), cognitive performance (DANA), balance measures (ClearEdge), and saliva from 150 children (7-21 years) with concussion over 5 time-points: 0-2, 3-6, 7-14, 15-29, and 30-60 days post-injury. Saliva miRNA levels within the 443 concussion samples were quantified with RNA sequencing and compared to 218 samples from age- and sex-matched controls (healthy and post-exercise participants). Non-parametric ANOVA assessed RNA levels across time-points, and between concussions/controls. Machine learning was used to build logistic regression algorithms differentiating concussions/controls, and symptomatic/recovered concussion participants. Relationships between miRNAs and concussion phenotypes were explored with Spearman’s Rank correlations. RESULTS/ANTICIPATED RESULTS: Fifteen miRNAs differed across control and concussion participants (FDR < 0.05). Within concussion participants, all 15 miRNAs trended back toward control levels by 30-60 days post injury. A regression algorithm employing 6 of the 15 miRNAs differentiated control and concussion participants with an area under the curve (AUC) of 0.78 in a training set (n = 244) and 0.84 in a naïve test set (n = 24). Similarly, 6 miRNAs were able to differentiate symptomatic (SCAT-5 symptom score > 7) and asymptomatic concussion participants with an AUC of 0.73 in a training set (n = 219) and 0.76 in a naïve test set (n = 44). Furthermore, 5 miRNAs showed significant (R > 0.3; FDR < 0.05) associations with subjective and/or objective measures of concussion-related symptoms. DISCUSSION/SIGNIFICANCE OF IMPACT: Saliva miRNAs levels are altered in children with concussion, and display predictable longitudinal trends following injury. Saliva miRNA measurement represents a non-invasive, objective tool that could be rapidly assessed to provide biologic evidence for clinicians managing pediatric concussion. CONFLICT OF INTEREST DESCRIPTION: I serve as a paid consultant and scientific advisory board member for Quadrant Biosciences, who has funded a portion of this work and licensed the findings from the Penn State College of Medicine.

